# Disruptions to routine childhood vaccinations in low- and middle-income countries during the COVID-19 pandemic: A systematic review

**DOI:** 10.3389/fped.2022.979769

**Published:** 2022-08-11

**Authors:** Alexandra M. Cardoso Pinto, Lasith Ranasinghe, Peter J. Dodd, Shyam Sundar Budhathoki, James A. Seddon, Elizabeth Whittaker

**Affiliations:** ^1^School of Medicine, Imperial College London, London, United Kingdom; ^2^Academic Foundation Doctor, Imperial College London, London, United Kingdom; ^3^School of Health and Related Research, University of Sheffield, Sheffield, United Kingdom; ^4^Department of Primary Care and Public Health, School of Public Health, Imperial College London, London, United Kingdom; ^5^Department of Infectious Disease, Imperial College London, London, United Kingdom; ^6^Desmond Tutu TB Centre, Department of Paediatrics and Child Health, Stellenbosch University, Cape Town, South Africa

**Keywords:** immunization, routine vaccines, LMICs, child health, vaccine-preventable diseases, vaccination hesitancy

## Abstract

**Background:**

The COVID-19 pandemic has disrupted routine childhood vaccinations worldwide with low- and middle-income countries (LMICs) most affected. This study aims to quantify levels of disruption to routine vaccinations in LMICs.

**Methods:**

A systematic review (PROSPERO CRD42021286386) was conducted of MEDLINE, Embase, Global Health, CINAHL, Scopus and MedRxiv, on the 11th of February 2022. Primary research studies published from January 2020 onwards were included if they reported levels of routine pediatrics vaccinations before and after March 2020. Study appraisal was performed using NHLBI tool for cross-sectional studies. Levels of disruption were summarized using medians and interquartile ranges.

**Results:**

A total of 39 cross-sectional studies were identified. These showed an overall relative median decline of −10.8% [interquartile range (IQR) −27.6%, −1.4%] across all vaccines. Upper-middle-income countries (upper-MICs) (−14.3%; IQR −24.3%, −2.4%) and lower-MICs (−18.0%; IQR −48.6%, −4.1%) showed greater declines than low-income countries (−3.1%; IQR −12.8%, 2.9%), as did vaccines administered at birth (−11.8%; IQR −27.7%, −3.5%) compared to those given after birth (−8.0%; IQR −28.6%, −0.4%). Declines during the first 3 months of the pandemic (−8.1%; IQR −35.1%, −1.4%) were greater than during the remainder of 2020 (−3.9%; IQR −13.0%, 11.4%) compared to baseline.

**Conclusion:**

There has been a decline in routine pediatric vaccination, greatest in MICs and for vaccines administered at birth. Nations must prioritize catch-up programs alongside public health messaging to encourage vaccine uptake.

**Systematic review registration:**

Identifier: CRD42021286386.

## Background

The Coronavirus Disease 2019 (COVID-19) pandemic (hereafter, “the pandemic”) and its control measures have disrupted access to healthcare globally. A systematic review performed during the first months of the pandemic found an overall 37% reduction in health service utilization, including hospital admissions, diagnostic and treatment services, highest during March and April 2020 (1). In May 2020, the World Health Organization (WHO) released the first Pulse Survey amongst Ministry of Health officials globally; nearly 90% reported disruptions to essential health services (2). Disruptions were greater in low-income countries (LICs) than high-income countries (HICs) (2). Immunization services were amongst those most frequently reported to be affected (2), with UNICEF estimating that 23 million children did not receive routine vaccinations during 2020; 3.7 million more than in 2019 (3).

Two further Pulse Surveys were published in May 2021 (4) and February 2022 (5). These showed that over 90% of countries reported continued healthcare disruptions. Of particular importance is the increased disruption to immunization services; whilst in May 2021 over one third of nations reported disruptions to immunization services (4), this rose to nearly half of nations in the subsequent survey (5). These findings raise concern regarding vaccine-preventable childhood morbidity and mortality. A modeling study by researchers at Johns Hopkins School of Public Health estimated a possible 9.8–44.7% increase in monthly deaths in children under-5 years caused by pandemic-related disruptions to healthcare, including vaccinations (6).

Routine vaccinations are fundamental for the health of children. A modeling study, investigating 10 pediatric vaccines, predicted that between 2000 and 2019, ~37 million deaths were prevented in low- and middle-income countries (LMICs) through vaccination (7). This represents a 45% decrease in mortality compared to a no-vaccine scenario, with most of the avoided deaths in children under 5 years (7). Most vaccines in this study are part of the WHO list of universally recommended immunizations, which include: Bacille Calmette-Guérin (BCG), Hepatitis B, Polio, diphtheria-tetanus-pertussis-containing (DTP) including Pentavalent, *Haemophilus influenzae* type b, Pneumococcal (conjugate), Rotavirus, Measles-containing (MCV), Rubella and Human Papillomavirus (HPV) vaccinations (8). Widespread access to these vaccines is essential to achieve universal health and wellbeing—part of Sustainable Development Goal (SDG) 3—in addition to other SDGs indirectly, including the reduction of poverty, malnutrition and achieving economic prosperity (9, 10). However, prior to the pandemic, the WHO had already highlighted large disparities in vaccine coverage worldwide. For example, in 2019, coverage of the third dose of DTP vaccine was only 73% in Africa, compared to 95% in Europe (11); inequalities which may widen with pandemic-related disruptions (9, 10, 12, 13).

Given a lower initial coverage of routine vaccinations, greater disruptions to healthcare during the pandemic, higher burden of vaccine-preventable diseases and lower available financial and infrastructural resources, LMICs are likely to encounter further challenges in the recovery of missed vaccinations (2, 4, 5, 11, 12). Gaining insight into the extent of pandemic-related disruptions to vaccination services is essential to plan effective catch-up vaccination programs, avoid vaccine-preventable disease epidemics and establish guidance to prevent disruptions in future global health emergencies. Therefore, the aim of this study is to measure the impact of the COVID-19 pandemic on routine childhood vaccination in LMICs.

## Methods

A systematic review of published and pre-print literature were performed.

### Search strategy

Six databases were searched: Medline, EMBASE and Global Health *via* Ovid, CINAHL, and Scopus. No field limits were applied. MedRxiv titles and abstracts were also searched, using the “medrxivr” package on R (14, 15). All searches were performed on the 11th of February 2022 and limited to publications from January 2020 onwards. The search strategy contained three concepts: COVID-19, immunization and specific vaccines or vaccine-preventable diseases (Supplementary Table S1). Additionally, a concept on general terms for routine vaccines was included, using proximity Boolean terms. This limited the number of irrelevant results, namely those related to COVID-19 vaccines. The search contained relevant keywords, including variations, and subject headings (Supplementary materials 2–7 contain full search strategies).

References of all relevant reviews, meeting and conference summaries, and all included studies, were screened for inclusion. Full-text versions of relevant abstracts were searched for in the previously mentioned databases and relevant journals. If unavailable, abstract authors were contacted to request access to full-texts.

### Inclusion and exclusion criteria

Primary research studies reporting the levels, or changes in levels, of vaccine coverage or administration before (any time between January 2015 to March 2020) and during the pandemic (March 2020 onwards) in LMICs were included. Studies had to include data for LMICs regarding any vaccine universally recommended by the WHO, published from 2020 onwards. Non-primary research and modeling studies, such as those predicting the impact of the pandemic on future vaccination levels without accompanying observed measurements, were excluded. Language restrictions were only applied at full-text stage; studies not in English, Portuguese, French or Spanish were translated to English using Google Translate. Studies were only excluded if the translation was unclear.

### Result screening and selection

Deduplication was performed on EndNote 20, and then Covidence, where screening was undertaken. Given the high number of identified studies, initial screening was performed by title to exclude clearly irrelevant results, followed by abstract. Eligibility was confirmed in full-text review. Screening was performed by two reviewers independently with discrepancies resolved by consensus.

### Data extraction and quality assessment

Data were extracted from included studies using a pre-defined data extraction sheet designed on Microsoft Excel, including the following parameters: publication details (doi, authors, title, year published), study details (design, scope, data source, sample size, location(s) of study, country income-level classification, population, sampling methods, funding, conflicts of interest), outcome of interest details (date span of data in pre-COVID and COVID periods, use of controls, vaccines included, outcome title and outcome units), results for each outcome of interest, methods of analysis and conclusions. Outcomes of interest included number of vaccines administered pre- and during COVID-19 pandemic; vaccine coverage—defined as the number of individuals receiving a certain vaccine as a percentage of the target population for that vaccine in a specific time-period—pre- and during COVID-19 pandemic; and proportional or percentage change in either outcome. Where available, outcomes pre- and during COVID-19 pandemic were extracted per smallest unit of time available, usually per month. Where data were only available in graphical format, WebPlotDigitizer 4.5 (16) was used for extraction.

Studies underwent quality and bias assessment using National Heart, Lung and Blood Institute (NHLBI) checklist for observational studies (17). Data from 8 randomly selected studies (20% of total) were extracted by two reviewers. Given that all data extracted was identical, the remaining extractions were performed by a single reviewer. Bias assessments were performed fully by two reviewers and discrepancies resolved by consensus.

### Data synthesis and analysis

As there are no universally-accepted guidelines for conducting systematic reviews and meta-analyses of proportional changes, a guide published in BMC Medical Research Methodology (18), the Cochrane Handbook for Systematic Reviews of Interventions (19) and COSMOS-E guidelines (20) were consulted and adapted as appropriate.

Although a meta-analysis was planned it was not performed because studies were found to have substantial methodological variation, including in the vaccines studied, scope of data and locations. Furthermore, only a minority of studies reported uncertainty levels and other data required for meta-analysis. Instead, studies were summarized using medians and interquartile ranges (IQRs). The outcome unit was mean relative percentage change between levels of vaccination pre-COVID-19 pandemic (from January 2015 to February 2020) and during the pandemic (April 2020 to December 2021). Where percentage changes were not reported, these were calculated using pre-pandemic and pandemic values. Timelines for each study varied according to availability of data (Supplementary Figure S2). March 2020 was excluded from studies that reported data per month as this was considered a transition point.

Subgroup analyses by timing of vaccination (birth or afterwards), individual routine vaccine, WHO world region and income-level were performed. Results were also subdivided by decline during the first 3-months of the pandemic (April-June 2020) and the remainder of the pandemic, to identify potential recovery. The data extraction sheet on Microsoft Excel was used to determine which studies could be included in each subgroup.

### Registration

This systematic review was registered on PROSPERO (CRD42021286386) and followed PRISMA 2020 guidelines (Supplementary Tables S3, S4) (21, 22).

### Protocol changes

Amendments to chosen databases were instituted after consultation with an expert librarian. This included the removal of Web of Science, as this had significant overlap with Scopus and the addition of MedRxiv for pre-prints.

## Results

**Figure 1:**
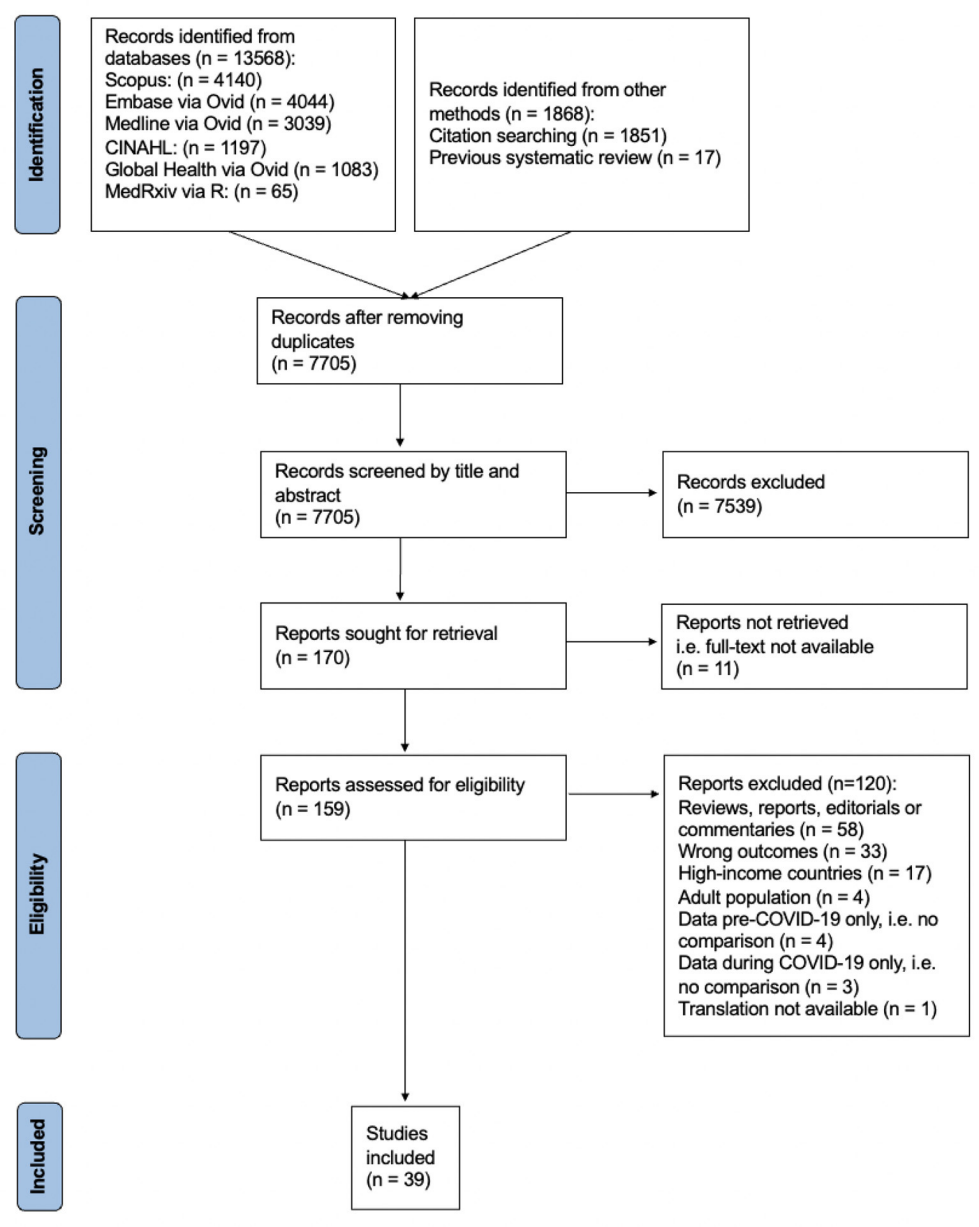
PRISMA flow diagram summarizing identification, screening, exclusion, and inclusion of studies (see Supplementary Figure 1 for detailed PRISMA 2020 flow diagram).

Following the screening of 7,705 studies, 39 were included in the review (Figure 1).

All studies were cross-sectional, utilizing data from health records (Table 1). Most reported levels of administered vaccines (*n* = 29) and the remainder reported vaccine coverage. Studies spanned 6 WHO regions unevenly, with Africa (53.8%) being the most common. Additionally, several countries appear repeatedly in different studies (Supplementary Table S2). Most studies reported national-level data (*n* = 17) or data from multiple health centers or regions (*n* = 11); the remainder were single-center studies and one survey-based study with unclear scope. Data were available for all WHO universally recommended vaccines apart from HPV, with levels of pentavalent or DTP (*n* = 33), MCV (*n* = 27) and BCG (*n* = 20) vaccines most frequently reported.

**Table 1:** Summary of study characteristics for studies reporting changes to vaccination levels (*n* = 39).

		**All identified studies**	
		**Number of studies (%)**	**Study references**
Study design	Cross sectional	39 (100.0)	(23–61)
			
WHO world region	African Region (AFR)	21 (53.8)	(23, 25, 26, 28–31, 33, 35, 37, 39, 40, 42, 46, 47, 52–55, 59, 61)
	Region of the Americas (AMR)	11 (28.2)	(24, 34–36, 38, 48, 51, 54, 57, 58, 60)
	Eastern Mediterranean Region (EMR)	8 (20.5)	(27, 32, 45, 49, 50, 53, 54, 56)
	South-East Asian Region (SEAR)	3 (7.7)	(43, 44, 54)
	Western Pacific Region (WPR)	1 (2.6)	(54)
	European Region (EUR)	1 (2.6)	(41)
Income level	Low-income countries (LICs)	14 (35.9)	(23, 25, 29, 31, 35, 37, 39, 42, 45–47, 49, 53, 61)
	Lower-middle-income countries (lower-MICs)	17 (43.6)	(26–30, 32, 33, 35, 43, 44, 46, 50, 52, 53, 55, 56, 59)
	Upper-middle-income countries (upper-MICs)	13 (33.3)	(24, 29, 34, 36, 38, 40, 41, 46, 48, 51, 57, 58, 60)
Scope of data	Multinational	1 (2.6)	(54)
	National	17 (43.6)	(24, 25, 28, 29, 34, 36, 38, 45, 46, 48, 51–53, 55, 57, 58, 61)
	Multicenter (national)	11 (28.2)	(23, 26, 27, 32, 35, 39–42, 49, 56)
	Single center (national)	9 (23.1)	(30, 31, 33, 37, 43, 44, 50, 59, 60)
	Unclear/NA	1 (2.6)	(47)
Data source	Health records/database (at government or local authority level)	25 (64.1)	(23–29, 32, 34, 36, 38–41, 45, 48, 49, 51–53, 55–58, 61)
	Health records/database (at hospital or medical center level)	9 (23.1)	(30, 31, 33, 42–44, 50, 59, 60)
	NGO records	2 (5.1)	(46, 54)
	Survey	1 (2.6)	(47)
	Unclear	2 (5.1)	(35, 37)
Vaccines	Pentavalent or Diphtheria-Tetanus-Pertussis vaccine (DTP)	33 (84.6)	(23–36, 38, 39, 41–46, 48–50, 52–55, 57, 58, 60, 61)
	Measles-containing vaccine (MCV)	27 (69.2)	(25–36, 38, 40–44, 46, 48, 50–52, 54, 57, 60, 61)
	Bacillus Calmette-Guérin vaccine (BCG)	20 (51.3)	(24, 26, 30–32, 34, 35, 38, 39, 41, 43, 44, 46, 48, 50, 52, 53, 57, 58, 61)
	Pneumococcal vaccine	12 (30.8)	(24–26, 31, 32, 34, 35, 38, 39, 48, 58, 61)
	Rotavirus vaccine	11 (28.2)	(24, 31, 32, 34, 35, 38, 43, 44, 48, 50, 61)
	Polio vaccine (any, including unspecified)	14 (35.9)	(24, 26, 31, 32, 34, 35, 39, 41, 43, 44, 48, 50, 57, 61)
	Oral polio vaccine (OPV)	9 (23.1)	(26, 31, 32, 39, 41, 43, 44, 50, 61)
	Inactivated poliovirus vaccine (IPV)	7 (17.9)	(26, 31, 43, 44, 48, 57, 61)
	Hepatitis B vaccine	9 (23.1)	(24, 26, 34, 41, 43, 44, 48, 50, 57)
	Multiple vaccines (i.e., reporting two or more vaccines combined)	11 (28.2)	(30, 32, 33, 37, 42, 43, 47, 51, 52, 56, 59)
Extracted outcomes	**Vaccine administration**	**29 (74.4)**	(23, 24, 27, 30–33, 35–40, 42–46, 49–56, 59–61)
	Observed values	12 (30.8)	(24, 30, 31, 33, 37, 45, 46, 51, 56, 59–61)
	Mean values	10 (25.6)	(23, 32, 38, 40, 42, 44, 49, 50, 52, 55)
	Percentage difference	9 (23.1)	(27, 30, 32, 36, 37, 40, 43, 50, 54)
	Adjusted percentage difference	3 (7.7)	(35, 51, 53)
	Other	2 (5.1)	(36, 39)
	**Vaccine coverage**	**10 (25.6)**	(25, 26, 28, 29, 34, 41, 47, 48, 57, 58)
	Observed values	7 (17.9)	(25, 28, 29, 47, 48, 57, 58)
	Mean values	2 (5.1)	(26, 34)
	Percentage difference	3 (7.7)	(29, 41, 47)
Comparison timeline	Same months^*^	23 (69.0)	(23–25, 29–31, 34, 37, 41–43, 45–49, 54, 55, 57–61)
	Different months	16 (41.0)	(26–28, 32, 33, 35, 36, 38–40, 44, 50–53, 56)

*See Supplementary Table B2.1 for individually reported study characteristics. The bold values indicate the different ways of reporting vaccine administration or vaccine coverage*.

* *Yearly coverage assumed to be same months, unless otherwise stated*.

Timelines varied across studies (Supplementary Figure S2), with the median timespan being January 2019 (IQR: December 2017–July 2019) to September 2020 (IQR: June 2020–November 2020).

**Figure 2:**
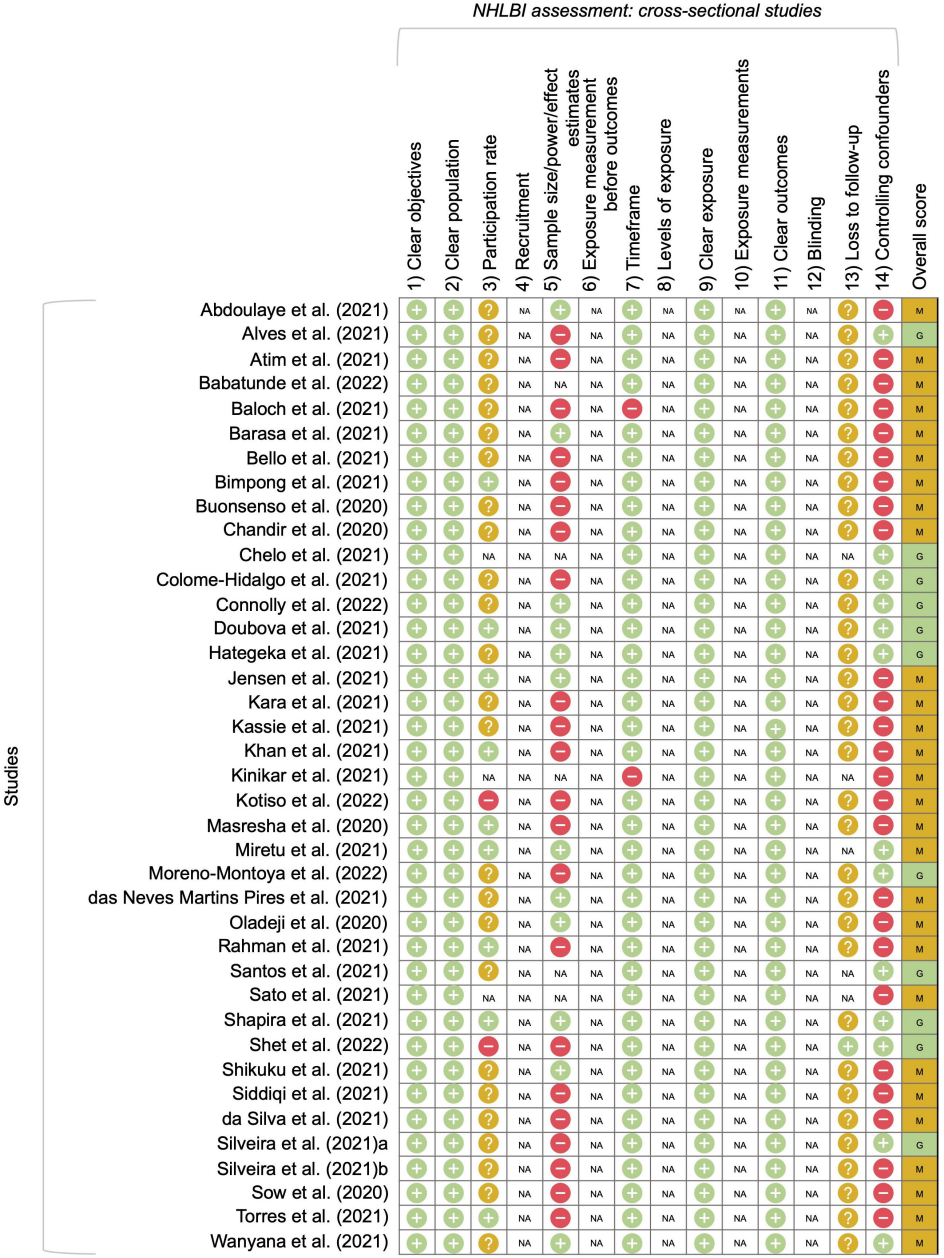
NHLBI assessments for included studies [Overall scores: good (G), moderate (M), poor (P)].

Overall, the quality of most studies was moderate; few studies considered confounders such as seasonality and population changes, and most did not report total population of the study or participation rates (Figure 2).

**Figure 3:**
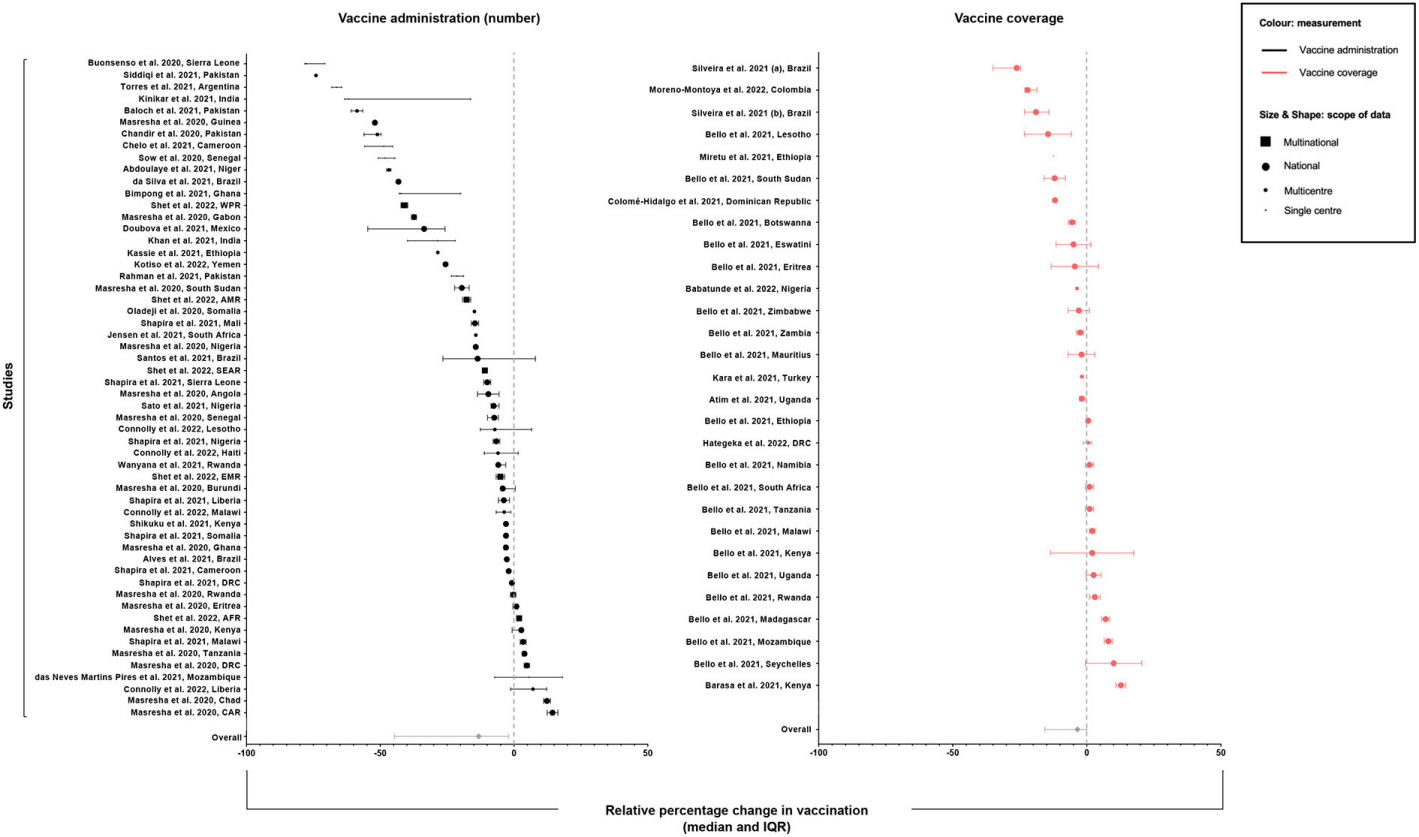
Median relative percentage change (±IQR) in vaccination coverage or number of vaccines administered per study and overall across all studies.

The overall median relative percentage change was −10.8% (IQR −27.6%, −1.4%) (Figure 3). This value was calculated using 331 observations, representing 45 countries (Table 2). The decline in studies reporting numbers of vaccines administered (−13.2%, IQR −44.7%, −2.0%) was greater than those reporting vaccination coverage (−3.5%, IQR −15.7%, 0.0%).

**Table 2:** Median change (± interquartile range) in level of vaccination.

	**Measurement**	***n* (observations)**	***n* (studies)**	*n* (countries)*	**Overall relative change [median]**	**Q1**	**Q3**
	Overall	331	39	50	−10.8	−27.6	−1.4
Outcome	Number of vaccines administered	236	28	38	−13.2	−44.7	−2.0
	Vaccine coverage	96	11	23	−3.5	−15.7	0.0
WHO world region	AFR	195	20	36	−4.0	−14.1	2.2
	AMR	59	10	7	−17.9	−24.3	−8.8
	SEAR	26	3	2	−28.6	−53.6	−18.4
	EMR	39	7	4	−34.5	−51.4	−19.1
	WPR	2	1	1	−41.0	−42.3	−39.7
	EUR	10	1	1	−1.9	−2.4	−1.2
Income level^*^	LIC	124	10	19	−3.1	−12.8	2.9
	Lower-MIC	130	16	14	−18.0	−48.6	−4.1
	Upper-MIC	67	12	12	−14.3	−24.3	−2.4
Vaccine age group	Birth	37	19	19	−11.8	−27.7	−3.5
	After birth (up to 2 years)	269	33	50	−8.2	−28.6	−0.4
Individual vaccines	BCG	27	16	18	−9.9	−23.0	−3.1
	Hep B	10	8	7	−7.5	−16.6	−1.6
	Polio	48	13	14	−16.6	−50.9	−3.9
	OPV	23	9	7	−28.6	−53.2	−6.0
	IPV	9	8	7	−26.2	−53.6	−21.7
	DTP/Penta	101	31	50	−7.4	−23.9	−0.1
	Rota	22	11	11	−22.4	−45.2	−6.9
	PCV	26	13	13	−4.7	−31.1	0.8
	MCV	80	27	46	−5.2	−21.2	1.7
Timeline	April to June 2020	91	19	28	−8.1	−35.1	−1.4
	June 2020 onwards	75	10	15	−3.9	−13	11.4

*AFR, African Region; AMR, Region of the Americas; SEAR, South-East Asian Region; EMR, Eastern Mediterranean Region; WPR, Western Pacific Region; EUR, European Region; LIC low-income country; lower-MIC, lower-middle-income country; upper-MIC, upper-middle-income country; BCG, Bacillus Calmette-Guérin vaccine; Hep B, Hepatitis B vaccine; OPV, oral polio vaccine; IPV, inactivated polio vaccine; DTP, diphtheria, tetanus and pertussis vaccine; Penta, pentavalent vaccine; Rota, rotavirus vaccine; PCV, pneumococcal conjugate vaccine; MCV, measles-containing vaccine*.

* *Shet et al. (54) not included income level analysis as this was a multinational study, reporting a single value per region. It was counted as a single country as the countries included in this study are not specified*.

**Figure 4:**
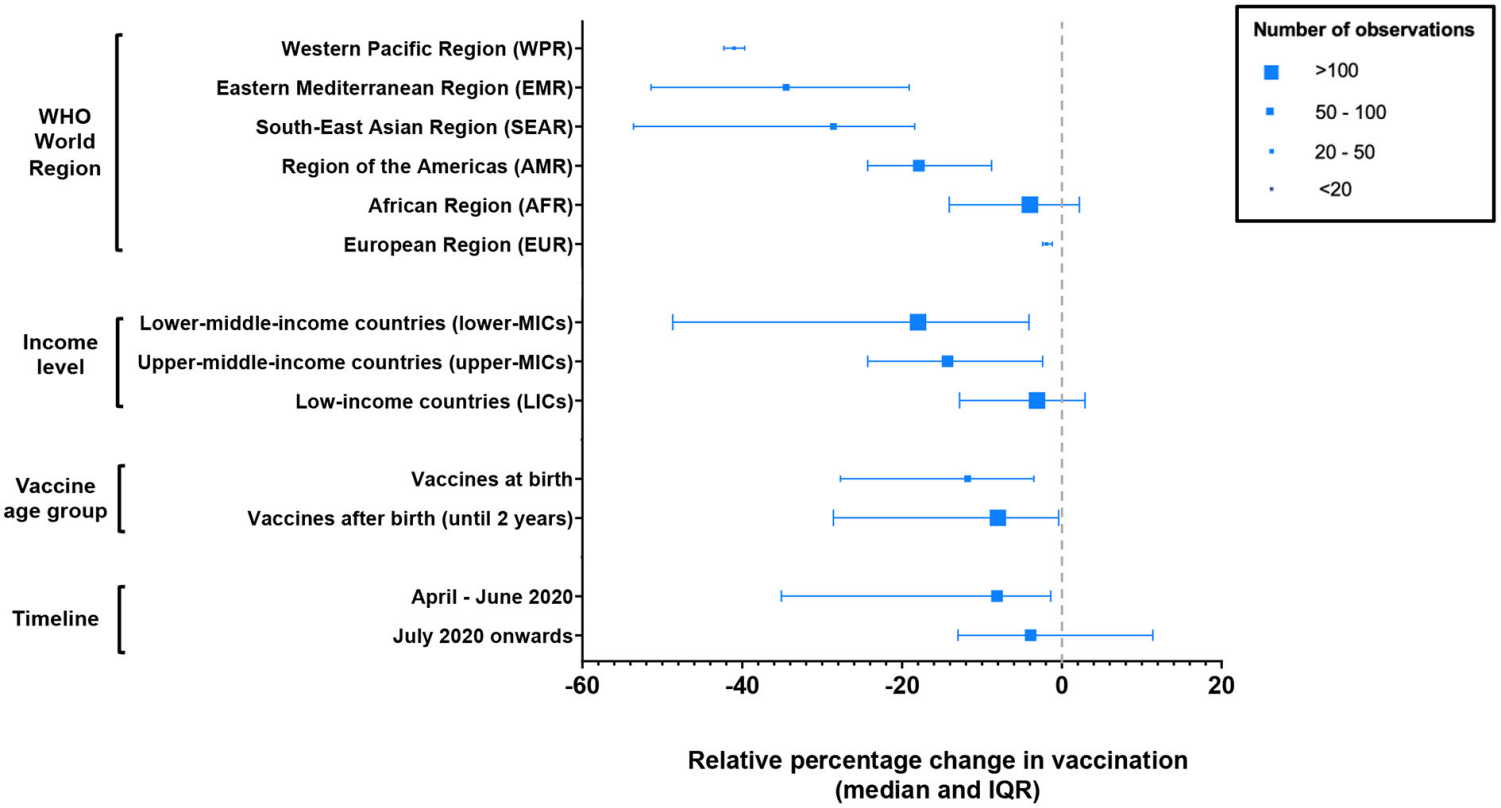
Median relative percentage change (±IQR) in vaccination by WHO world region, income level and vaccine age group.

The median decline was greater in upper-middle income countries (MICs) (−14.3%, IQR −24.3%, −2.4%) and lower-MICs (−18.0%, IQR −48.6%, −4.1%) than LICs (−3.1%, IQR −12.8%, 2.9%) (Figure 4). There were 19 (70.4%) LICs represented in this analysis, compared to 12 (21.8%) and 14 (25.5%) upper-MICs and lower-MICs, respectively.

The WHO world regions showing the greatest declines were WPR (−41.0%; IQR −42.3%, −39.7%), EMR (−34.5%, IQR −51.4%, −19.1%) and SEAR (−28.6%, IQR −53.6%, −18.4%). Regions showing the least declines were EUR (−1.9%, IQR −2.4%, −1.2%), followed by AFR (−4.0%, IQR −14.1%, 2.2%). However, whilst 35 countries from the AFR region were included in this analysis, the remaining regions had 6 or fewer represented countries [excluding Shet et al. (54)]. The study by Shet et al. (54) is an observational study summarizing global WHO vaccination coverage data which was included in the analysis for AFR, AMR, EMR, SEAR and WPR regions.

Vaccines administered at birth showed a median decline of −11.8% (IQR −27.7%, −3.5%) and vaccines after birth a decline of −8.2% (IQR −28.8%, −0.6%). Vaccines showing the greatest degrees of decline were polio vaccines (−16.6%, IQR −50.9%, −3.9%) and rotavirus vaccines (−22.4%, IQR −45.2%, −6.9%). Those showing the least declines were PCV (−4.7%, IQR −31.1%, 0.8%), followed by MCV (−5.2%, IQR −21.2%, 1.7%) and DTP or pentavalent vaccines (−7.4%, IQR −23.9%, −0.1%).

Declines during the first three months of the pandemic, that is April to June 2020, were greater (−8.1%, IQR −35.1%, −1.4%) than declines during the remainder of the pandemic relative to baseline, for time periods available (−3.9, −13.0, 11.4%).

## Discussion

Overall, a median decline of over 10% was seen in routine childhood vaccination in LMICs. Most countries represented in the analysis were from the WHO African region. Drops were greatest for vaccines given at birth, and in MICs. The drop in the first 3 months of the pandemic appears greater than later in the pandemic, suggesting a degree of recovery, although declines persist.

The decline in vaccination coverage corroborates findings from a previous systematic review which narratively synthesized evidence from LMICs and HICs in early 2021 (62), and the three WHO Pulse Surveys (2, 4, 5), all of which identified global disruptions to routine vaccination programs. The second Pulse Survey categorized results by income-level, also demonstrating that a greater proportion of MICs reported disruptions than LICs (4). Reasons for this are unclear, but maybe a consequence of publication bias, particularly as fewer MICs are represented in WHO data, and this study, than LICs. Alternative reasons to be explored include differences in stringency of COVID-19 measures, support from non-governmental organizations, such as the Global Alliance for Vaccines and Immunization (GAVI), and degrees of urbanization, particularly if these areas are found to have been more affected than rural areas.

This study found evidence that the median decline in vaccination during the first 3 months of 2020 was greater than the decline in the remainder of that year. This suggests there may have been some recovery in vaccination levels since the start of the pandemic, but declines persist. This corroborates findings from the third Pulse Survey; 53% of countries that participated in all three survey rounds reported disruptions to immunizations, compared to 56% in the first round, suggesting little improvement (5). By contrast, literature from England (63), France (64), Sweden (65), Japan (66) and the United States (67) suggests vaccination is recovering in these HICs, although not always returning to pre-pandemic levels. Data from these studies are from 2020; more recent data are needed for definitive conclusions on recovery. One study from Sierra Leone has since published data on vaccination declines until March 2021 by quarter. This dynamic analysis showed that despite improvements in vaccination levels in every quarter, most vaccines continued to show declines of over 10% by March 2021 (68). If recovery is greater in HICs than LMICs, these findings raise concern over potential widening of global inequalities in vaccination (13).

Given WHO recommendations to continue vaccination during the pandemic (69, 70), disruptions to maternal health services may explain part of the observed decline in vaccines delivered at birth. Observational studies from Bangladesh (71) and Nepal (72) have shown reductions in institutional deliveries of 10–20% and over 50%, respectively, during the first 3 months of the pandemic. By the end of 2021, 26% of countries still reported a decline in facility-based births to the WHO (5). The BCG vaccine was also thoroughly investigated for its use against COVID-19 (73), which may have led to temporary shortages in its supply, as was reported in Japan (74, 75). It is possible, however, that the finding that vaccines delivered in hospital soon after birth fell more than vaccines given in primary care later in infancy, is a result of the way data are collected and reported, or a function of the different studies included in this review.

The reasons behind disruptions to vaccinations are likely multifactorial. WHO findings suggest that 76% of reasons underlying disruptions to health-services stem from disruptions to healthcare service provision (5). A multinational study of IMPRINT members also identified fear of COVID-19 as a reason for delayed vaccination (76). Reasons for disruption are likely to vary according to each country's experience of the pandemic, including public health messaging and lockdown measures.

Vaccine hesitancy may also have contributed to declines in vaccination. Although vaccine hesitancy existed prior to COVID-19, hesitancy may have been exacerbated by the pandemic. A Norwegian study investigated factors associated with vaccine hesitancy during the COVID-19 pandemic and found that the greatest predictors of hesitancy were perceived risks of vaccinations and preference for natural immunity (77). Trust for information shared by health officials appeared to reduce risk of hesitancy (77). However, in instances where health professionals are themselves unsure of vaccine safety—as happened with COVID-19 vaccination—and share this publicly, such as through social media, trust in healthcare professionals might instead increase hesitancy. Similarly, government messaging discouraging vaccination, as was seen in Brazil with regards to COVID-19 vaccination (78), also has potential to translate into hesitancy across other vaccines.

Declines in routine childhood vaccination raise concern over future morbidity and mortality of vaccine-preventable diseases. Prior to the pandemic, many LMICs already had rates of vaccination coverage below the levels necessary to eliminate these diseases or achieve herd immunity (11). Such setbacks bring nations further away from achieving these targets. A modeling study predicted that an 18.5% decline in routine child vaccinations would result in a 10% increase in severely malnourished children, with declines in WHO universally recommended vaccines independently responsible for ~15 thousand additional deaths every three months (6). An older modeling study investigating the impact of falls in BCG coverage estimated that a 10% annual decline in BCG coverage worldwide could lead to over 11,700 tuberculosis deaths in children up to 15 years old (79).

It is not the first time that a disease outbreak has impacted healthcare. A systematic review found a decline in children's health services, including over 20% in pentavalent vaccinations, during the West Africa Ebola outbreak in 2014–2016 (80). Given its high transmissibility (81), the risk of measles outbreaks following declines in vaccination is particularly concerning; Guinea, Liberia and Sierra Leone all had significant rises in measles cases for up to 2 years following the Ebola outbreak (82). Vaccine-preventable disease outbreaks have already been reported during the pandemic for measles and polio (83, 84), including a polio outbreak in Malawi reported in February 2022 (85). Wild poliovirus was eliminated in Africa in 2020 (86); this outbreak brings major setbacks to polio eradication. Declines in vaccination are likely to increase the frequency and severity of these outbreaks. The fall in rotavirus vaccination is also concerning, as diarrhea has been reported as the second most common cause of death in children aged under five, excluding neonates, globally (87)—with rotavirus being the most common cause of severe or fatal diarrhea (88).

Furthermore, declines in surveillance and treatment have also been observed; over half of African countries reported reductions to suspected measles cases and lab specimens in 2020 (89). Whilst lockdown measures including school closures may have reduced transmission, considering the increasing trend in suspected measles cases between 2017 and 2019, declines are likely consequences of under-reporting (89). The combination of declines in vaccination with reduced healthcare-seeking behavior and less robust surveillance raise concern over increased prevalence, transmission, and severity of infections.

Efforts to recover lost vaccinations, such as catch-up programs, should be prioritized. Additionally, it is vital for nations to invest in public health campaigns encouraging attendance to essential health-services, including vaccinations. National investigations exploring factors disrupting vaccination programs and the extent of disruption for individual vaccines should also be performed, to ensure targeted approaches to catch-up programs. There may also be regional differences to investigate (77). These data would enable the prioritization of populations and vaccines with the highest level of disruption and risk of transmission. Greater understanding would also enable the development of guidance to prevent similar disruptions in future pandemics.

There are several limitations that should be acknowledged. First, given the substantial methodological heterogeneity between studies and missing participation rates for most studies, a meta-analysis was not performed. The analysis is descriptive, and measures of effect must be interpreted with caution. In addition, there is lack of representation from several world regions, with most studies reporting data from African countries. Similarly, there is low representation of MICs. Furthermore, available data is mainly from 2020; more recent data is required to establish reliable conclusions. These data limitations emphasize the need for recent national-level data from more countries and per vaccine, to improve the generalizability of findings and inform more meaningful analyses, respectively. Moreover, studies measuring levels of vaccine administration and coverage were included and assumed equal; however, this assumes that there is no change in population from pre-pandemic to pandemic time-periods. Most studies also did not account for confounders such as seasonality or secular trends. Finally, this study did not explore reasons behind disruptions to vaccination, including the potential impact of vaccination hesitancy during the COVID-19 pandemic.

Overall, this study found a drop in routine childhood vaccination in LMICs during the COVID-19 pandemic, with some evidence of recovery in 2020. To avoid increases in child mortality due to the resurgence of vaccine-preventable diseases, LMICs must now focus on recovery of lost vaccination through catch-up programs and strong public health messaging to encourage attendance to health services for routine vaccinations.

## Data availability statement

The original contributions presented in the study are included in the article/Supplementary material, further inquiries can be directed to the corresponding author/s.

## Author contributions

AC, JS, and EW designed the study and protocol. AC conducted first round of study screening, data extraction, and appraisal, conducted data analysis, and wrote the first draft of the manuscript. LR conducted second round of study screening, data extraction, and appraisal. JS and EW supervised the work. All authors provided critical feedback that helped shape the research, discussed results and contributed to the final version of the manuscript.

## Funding

Open access publication fees for this manuscript were covered by the Imperial College Open Access Fund. JS was supported by a Clinician Scientist Fellowship jointly funded by the UK Medical Research Council (MRC) and the UK Department for International Development (DFID) under the MRC/DFID Concordat agreement (MR/R007942/1).

## Conflict of interest

The authors declare that the research was conducted in the absence of any commercial or financial relationships that could be construed as a potential conflict of interest.

## Publisher's note

All claims expressed in this article are solely those of the authors and do not necessarily represent those of their affiliated organizations, or those of the publisher, the editors and the reviewers. Any product that may be evaluated in this article, or claim that may be made by its manufacturer, is not guaranteed or endorsed by the publisher.
