# Supplementary material for: Disruptions to routine childhood vaccinations in low- and middle-income countries during the COVID-19 pandemic: A systematic review
Source: Front Pediatr. 2022 Aug 11;10:979769. doi: 10.3389/fped.2022.979769 (PMC9403570; doi:10.3389/fped.2022.979769)
Supplement: Supplementary file 1 [file Table_1.DOCX]

**Table S.1** *–* Summary of search strategy with up to two keyword and subject heading examples*

| *Domain* | COVID-19 | *AND* | [General vaccine terms | *OR* | (Immunisation | *AND* | Specific vaccine terms)] |
| --- | --- | --- | --- | --- | --- | --- | --- |
| *Keywords* | COVID-19  *OR*  Coronavirus |  | Child* ADJ3 Vaccin*  *OR*  Routine ADJ3 immuni?ation* |  | Vaccin*  *OR*  Immuni?ation |  | Pneumococc*  *OR*  MMR* |
| *OR* | | | | | | | |
| *Subject Headings* | COVID-19/  *OR*  SARS-CoV-2/ |  |  |  | Vaccination/  *OR*  Immunization programs/ |  | Pneumococcal vaccines/  *OR*  Measles vaccine/ |

**Full search strategies found in sections A.2-A.7, reported as per PRISMA 2020 guidelines^(21,22)^.*
