# Supplementary material for: Disruptions to routine childhood vaccinations in low- and middle-income countries during the COVID-19 pandemic: A systematic review
Source: Front Pediatr. 2022 Aug 11;10:979769. doi: 10.3389/fped.2022.979769 (PMC9403570; doi:10.3389/fped.2022.979769)
Supplement: Supplementary file 2 [file Table_2.DOCX]

***S.2 - Search strategy for Ovid MEDLINE(R)***

1. COVID*.mp. (220555)

2. Sars-cov-2.mp. (145038)

3. pandemic.mp. (129153)

4. coronavirus.mp. (120424)

5. lockdown*.mp. (12239)

6. (Child* adj3 Vaccin*).mp. (16080)

7. (Child* adj3 Immuni?ation*).mp. (5789)

8. (Routine adj3 Immuni?ation*).mp. (2904)

9. (Routine adj3 vaccin*).mp. (3965)

10. (Infant adj3 Immuni?ation*).mp. (893)

11. (Infant adj3 vaccin*).mp. (1485)

12. (P?ediatric* adj3 Immuni?ation*).mp. (631)

13. (P?ediatric* adj3 vaccin*).mp. (1645)

14. MMR*.mp. (11372)

15. MCV*.mp. (6479)

16. Vaccin* program*.mp. (10497)

17. Immuni?ation* program*.mp. (16725)

18. (Vaccin* adj3 schedule*).mp. (4073)

19. (Immuni?ation* adj3 schedule*).mp. (13162)

20. vaccin*.mp. (428758)

21. immuni?ation*.mp. (172897)

22. BCG.mp. (31176)

23. polio*.mp. (37174)

24. DTP*.mp. (25272)

25. TDAP*.mp. (967)

26. diphtheria.mp. (21600)

27. whooping cough.mp. (9966)

28. pertussis.mp. (31304)

29. tetanus.mp. (29932)

30. pneumococ*.mp. (36673)

31. meningococ*.mp. (17197)

32. measles*.mp. (29371)

33. mumps.mp. (11637)

34. rubella.mp. (16685)

35. HPV*.mp. (49425)

36. papillomavirus.mp. (53804)

37. rota*.mp. (251228)

38. h?emophilus.mp. (30755)

39. pentavalent.mp. (2783)

40. hexavalent.mp. (5597)

41. PCV*.mp. (11409)

42. RCV*.mp. (1933)

43. Hepatitis B.mp. (106018)

44. Hep?B.mp. (3445)

45. IPV*.mp. (8029)

46. OPV*.mp. (3070)

47. HiB*.mp. (17511)

48. COVID-19/ (139194)

49. Pandemics/ (77838)

50. SARS-CoV-2/ (111080)

51. vaccination/ (92576)

52. immunization/ (52600)

53. immunization programs/ (12193)

54. immunization schedule/ (11303)

55. mass vaccination/ (3429)

56. vaccines, combined/ (2657)

57. Measles-mumps-rubella vaccine/ (2985)

58. Measles vaccine/ (7118)

59. Rubella vaccine/ (3051)

60. Mumps vaccine/ (1671)

61. BCG vaccine/ (19940)

62. Hepatitis B Vaccines/ (9740)

63. Poliovirus vaccines/ (1654)

64. Diphtheria-Tetanus-Pertussis Vaccine/ (3019)

65. Pneumococcal vaccines/ (8262)

66. Papillomavirus vaccines/ (9026)

67. Rotavirus vaccines/ (2692)

68. Haemophilus vaccines/ (3096)

69. Meningococcal vaccines/ (3731)

70. exp Poliovirus Vaccines/ (7716)

71. 1 or 2 or 3 or 4 or 5 or 48 or 49 or 50 (270486)

72. 6 or 7 or 8 or 9 or 10 or 11 or 12 or 13 or 16 or 17 or 18 or 19 (55229)

73. 20 or 21 or 51 or 52 or 53 or 54 or 55 (507587)

74. 14 or 15 or 22 or 23 or 24 or 25 or 26 or 27 or 28 or 29 or 30 or 31 or 32 or 33 or 34 or 35 or 36 or 37 or 38 or 39 or 40 or 41 or 42 or 43 or 44 or 45 or 46 or 47 or 56 or 57 or 58 or 59 or 60 or 61 or 62 or 63 or 64 or 65 or 66 or 67 or 68 or 69 or 70 (737449)

75. 73 and 74 (140220)

76. 72 or 75 (164450)

77. 71 and 76 (4288)

78. limit 77 to yr="2020 -Current" (3039)

***S.3 - Search strategy for Embase***

1. COVID*.mp. (219161)

2. Sars-cov-2.mp. (82443)

3. pandemic.mp. (162419)

4. coronavirus.mp. (237365)

5. lockdown*.mp. (13734)

6. (Child* adj3 Vaccin*).mp. (20903)

7. (Child* adj3 Immuni?ation*).mp. (6947)

8. (Routine adj3 Immuni?ation*).mp. (3378)

9. (Routine adj3 vaccin*).mp. (4953)

10. (Infant adj3 Immuni?ation*).mp. (1039)

11. (Infant adj3 vaccin*).mp. (1814)

12. (P?ediatric* adj3 Immuni?ation*).mp. (740)

13. (P?ediatric* adj3 vaccin*).mp. (2258)

14. MMR*.mp. (22620)

15. MCV*.mp. (12393)

16. Vaccin* program*.mp. (12922)

17. Immuni?ation* program*.mp. (8334)

18. (Vaccin* adj1 schedule*).mp. (4280)

19. (Immuni?ation* adj1 schedule*).mp. (3640)

20. vaccin*.mp. (584353)

21. immuni?ation*.mp. (219782)

22. BCG.mp. (63038)

23. polio*.mp. (55592)

24. DTP*.mp. (22100)

25. TDAP*.mp. (1380)

26. diphtheria.mp. (36148)

27. whooping cough.mp. (5095)

28. pertussis.mp. (48660)

29. tetanus.mp. (47446)

30. pneumococ*.mp. (49157)

31. meningococ*.mp. (22578)

32. measles*.mp. (46332)

33. mumps.mp. (21166)

34. rubella.mp. (28324)

35. HPV*.mp. (67977)

36. papillomavirus.mp. (63210)

37. rota*.mp. (304912)

38. h?emophilus.mp. (50738)

39. pentavalent.mp. (3404)

40. hexavalent.mp. (5639)

41. PCV*.mp. (15610)

42. RCV*.mp. (2846)

43. Hepatitis B.mp. (178363)

44. Hep?B.mp. (4990)

45. IPV*.mp. (9205)

46. OPV*.mp. (3018)

47. HiB*.mp. (25233)

48. coronavirus disease 2019/ (183811)

49. pandemic/ (108093)

50. Severe acute respiratory syndrome coronavirus 2/ (55616)

51. lockdown/ (7640)

52. national lockdown/ (664)

53. measles vaccination/ (3929)

54. diphtheria pertussis tetanus vaccine/ (9103)

55. immunization/ (118847)

56. measles mumps rubella vaccine/ (7914)

57. mass immunization/ (4715)

58. rubella vaccine/ (4845)

59. mumps vaccine/ (2688)

60. hepatitis B vaccine/ (20473)

61. poliomyelitis vaccine/ (11116)

62. Pneumococcus vaccine/ (21577)

63. Wart virus vaccine/ (15587)

64. Rotavirus vaccine/ (6124)

65. Haemophilus influenzae type b vaccine/ (5156)

66. Meningococcus vaccine/ (7853)

67. vaccination coverage/ (3779)

68. vaccination/ (185943)

69. BCG vaccine/ (46398)

70. 1 or 2 or 3 or 4 or 5 or 48 or 49 or 50 or 51 or 52 (309871)

71. 6 or 7 or 8 or 9 or 10 or 11 or 12 or 13 or 16 or 17 or 18 or 19 (53366)

72. 20 or 21 or 55 or 57 or 67 or 68 (677992)

73. 14 or 15 or 22 or 23 or 24 or 25 or 26 or 27 or 28 or 29 or 30 or 31 or 32 or 33 or 34 or 35 or 36 or 37 or 38 or 39 or 40 or 41 or 42 or 43 or 44 or 45 or 46 or 47 or 54 or 56 or 58 or 59 or 60 or 61 or 62 or 63 or 64 or 65 or 66 or 69 (1009121)

74. 72 and 73 (225687)

75. 71 or 74 (245740)

76. 70 and 75 (6065)

77. limit 76 to yr="2020 -Current" (4044)

***S.4 - Search strategy for Global Health***

1. COVID*.mp. (58500)

2. Sars-cov-2.mp. (28975)

3. pandemic.mp. (45452)

4. coronavirus.mp. (68145)

5. lockdown*.mp. (4390)

6. (Child* adj3 Vaccin*).mp. (10039)

7. (Child* adj3 Immuni?ation*).mp. (3608)

8. (Routine adj3 Immuni?ation*).mp. (2166)

9. (Routine adj3 vaccin*).mp. (2606)

10. (Infant adj3 Immuni?ation*).mp. (564)

11. (Infant adj3 vaccin*).mp. (900)

12. (P?ediatric* adj3 Immuni?ation*).mp. (228)

13. (P?ediatric* adj3 vaccin*).mp. (822)

14. MMR*.mp. (2602)

15. MCV*.mp. (2103)

16. Vaccin* program*.mp. (6469)

17. Immuni?ation* program*.mp. (8035)

18. (Vaccin* adj1 schedule*).mp. (1957)

19. (Immuni?ation* adj1 schedule*).mp. (1540)

20. vaccin*.mp. (150582)

21. immuni?ation*.mp. (93348)

22. BCG.mp. (6307)

23. polio*.mp. (8045)

24. DTP*.mp. (1621)

25. TDAP*.mp. (542)

26. diphtheria.mp. (5464)

27. whooping cough.mp. (5911)

28. pertussis.mp. (7518)

29. tetanus.mp. (7392)

30. pneumococ*.mp. (11249)

31. meningococ*.mp. (7293)

32. measles*.mp. (13098)

33. mumps.mp. (4303)

34. rubella.mp. (5663)

35. HPV*.mp. (19634)

36. papillomavirus.mp. (18851)

37. rota*.mp. (19780)

38. h?emophilus.mp. (9707)

39. pentavalent.mp. (1605)

40. hexavalent.mp. (764)

41. PCV*.mp. (4808)

42. RCV*.mp. (209)

43. Hepatitis B.mp. (40671)

44. Hep?B.mp. (784)

45. IPV*.mp. (2762)

46. OPV*.mp. (1297)

47. HiB*.mp. (4769)

48. severe acute respiratory syndrome coronavirus/ (6307)

49. pandemics/ (37114)

50. immunization/ (83777)

51. immunization programmes.sh. (5391)

52. mandatory vaccination/ (178)

53. vaccination/ (78395)

54. diphtheria pertussis tetanus vaccines/ (1855)

55. diphtheria tetanus pertussis poliomyelitis vaccines/ (21)

56. poliomyelitis vaccines/ (209)

57. Haemophilus influenzae vaccines/ (45)

58. combined vaccines/ (1161)

59. measles mumps rubella vaccines/ (1617)

60. pertussis vaccines/ (107)

61. BCG vaccine/ (3398)

62. 1 or 2 or 3 or 4 or 5 or 48 or 49 (84594)

63. 6 or 7 or 8 or 9 or 10 or 11 or 12 or 13 or 16 or 17 or 18 or 19 (27032)

64. immunization programmes/ (5391)

65. 20 or 21 or 50 or 52 or 53 or 64 (161955)

66. 14 or 15 or 23 or 24 or 25 or 26 or 27 or 28 or 29 or 30 or 31 or 32 or 33 or 34 or 35 or 36 or 37 or 38 or 39 or 40 or 41 or 42 or 43 or 44 or 45 or 46 or 47 or 54 or 55 or 56 or 57 or 58 or 59 or 60 or 61 (147223)

67. 65 and 66 (52829)

68. 63 or 67 (63095)

69. 62 and 68 (1950)

70. limit 69 to yr="2020 -Current" (1083)

***S.5 - Search strategy for Scopus***

( ( TITLE-ABS-KEY ( covid* ) )  OR  ( TITLE-ABS-KEY ( sars-cov-2 ) )  OR  ( TITLE-ABS-KEY ( pandemic ) )  OR  ( TITLE-ABS-KEY ( coronavirus ) )  OR  ( TITLE-ABS-KEY ( lockdown* ) ) )  AND  ( ( ( TITLE-ABS-KEY ( child*  W/2  vaccin* ) )  OR  ( TITLE-ABS-KEY ( child*  W/2  immuni?ation* ) )  OR  ( TITLE-ABS-KEY ( infant  W/2  immuni?ation* ) )  OR  ( TITLE-ABS-KEY ( infant  W/2  vaccin* ) )  OR  ( TITLE-ABS-KEY ( p?ediatric*  W/2  immuni?ation* ) )  OR  ( TITLE-ABS-KEY ( p?ediatric*  W/2  vaccin* ) )  OR  ( TITLE-ABS-KEY ( newborn  W/2  immuni?ation* ) )  OR  ( TITLE-ABS-KEY ( newborn  W/2  vaccin* ) )  OR  ( TITLE-ABS-KEY ( routine*  W/2  vaccin* ) )  OR  ( TITLE-ABS-KEY ( routine*  W/2  immuni?ation* ) )  OR  ( TITLE-ABS-KEY ( schedule*  W/2  vaccin* ) )  OR  ( TITLE-ABS-KEY ( schedule*  W/2  immuni?ation* ) )  OR  ( TITLE-ABS-KEY ( “vaccin*  program* “) )  OR  ( TITLE-ABS-KEY (“ immuni?ation*  program* “) ) )  OR  ( ( ( TITLE-ABS-KEY ( vaccin* ) )  OR  ( TITLE-ABS-KEY ( immuni?ation* ) ) )  AND  ( ( TITLE-ABS-KEY ( bcg ) )  OR  ( TITLE-ABS-KEY ( polio* ) )  OR  ( TITLE-ABS-KEY ( ipv* ) )  OR  ( TITLE-ABS-KEY ( opv* ) )  OR  ( TITLE-ABS-KEY ( dtp* ) )  OR  ( TITLE-ABS-KEY ( tdap* ) )  OR  ( TITLE-ABS-KEY ( diphtheria ) )  OR  ( TITLE-ABS-KEY ( pentavalent ) )  OR  ( TITLE-ABS-KEY ( hexavalent ) )  OR  ( TITLE-ABS-KEY ( whooping  AND cough ) )  OR  ( TITLE-ABS-KEY ( pertussis ) )  OR  ( TITLE-ABS-KEY ( tetanus ) )  OR  ( TITLE-ABS-KEY ( pneumococ* ) )  OR  ( TITLE-ABS-KEY ( pcv* ) )  OR  ( TITLE-ABS-KEY ( meningococ* ) )  OR  ( TITLE-ABS-KEY ( measles* ) )  OR  ( TITLE-ABS-KEY ( mcv* ) )  OR  ( TITLE-ABS-KEY ( mumps ) )  OR  ( TITLE-ABS-KEY ( rubella ) )  OR  ( TITLE-ABS-KEY ( rcv* ) )  OR  ( TITLE-ABS-KEY ( mmr* ) )  OR  ( TITLE-ABS-KEY ( hpv* ) )  OR  ( TITLE-ABS-KEY ( papillomavirus ) )  OR  ( TITLE-ABS-KEY ( rota* ) )  OR  ( TITLE-ABS-KEY ( h?emophilus ) )  OR  ( TITLE-ABS-KEY ( hib* ) )  OR  ( TITLE-ABS-KEY (“ hepatitis  b “) )  OR  ( TITLE-ABS-KEY ( hep?b ) ) ) ) )  AND  ( LIMIT-TO ( PUBYEAR ,  2022 )  OR  LIMIT-TO ( PUBYEAR ,  2021 )  OR  LIMIT-TO ( PUBYEAR ,  2020 ) )

***S.6 - Search strategy for CINAHL***

1. (MH "COVID-19 Pandemic")

2. (MH "SARS-CoV-2")

3. (MH "Vaccination Coverage")

4. (MH "Immunization")

5. (MH "Immunization Programs")

6. (MH "Immunization Schedule")

7. (MH "Vaccines, Combined")

8. (MH "BCG Vaccine")

9. (MH "Meningococcal Vaccines")

10. (MH "Diphtheria-Tetanus-Pertussis Vaccine")

11. (MH "Diphtheria-Tetanus Vaccine")

12. (MH "Diphtheria-Tetanus-acellular Pertussis Vaccines")

13. (MH "Measles-Mumps-Rubella Vaccine")

14. (MH "Measles Vaccine")

15. (MH "Rubella Vaccine")

16. (MH "Hepatitis B Vaccines")

17. (MH "Poliovirus Vaccine+")

18. (MH "Pneumococcal Vaccine")

19. (MH "Meningococcal Vaccines")

20. (MH "Papillomavirus Vaccine")

21. (MH "Rotavirus Vaccines")

22. (MH "HIB Vaccine")

23. COVID*

24. Sars-cov-2

25. Pandemic

26. Coronavirus

27. Lockdown*

28. Child* N2 Vaccin*

29. Child* N2 Immuni#ation*

30. Infant N2 Immuni#ation*

31. Infant N2 vaccin*

32. P#ediatric* N2 Immuni#ation*

33. P#ediatric* N2 vaccin*

34. newborn N2 Immuni#ation*

35. newborn N2 vaccin*

36. Routine* N2 vaccin*

37. Routine* N2 immuni#ation*

38. Schedule* N2 vaccin*

39. Schedule* N2 immuni#ation*

40. Vaccin* program*

41. immuni#ation* program*

42. Vaccin*

43. Immuni#ation*

44. BCG

45. Polio*

46. IPV*

47. OPV*

48. DTP*

49. TDAP*

50. Diphtheria

51. Pentavalent

52. Hexavalent

53. Whooping cough

54. Pertussis

55. Tetanus

56. Pneumococ*

57. PCV*

58. Meningococ*

59. Measles*

60. MCV*

61. Mumps

62. Rubella

63. RCV*

64. MMR*

65. HPV*

66. Papillomavirus

67. Rota*

68. haemophilus

69. HIB*

70. Hepatitis B

71. Hep#B

72. S1 OR S2 OR S23 OR S24 OR S25 OR S26 OR S27

73. S28 OR S29 OR S30 OR S31 OR S32 OR S33 OR S34 OR S35 OR S36 OR S37 OR S38 OR S39 OR S40 OR S41

74. S3 OR S4 OR S5 OR S6 OR S42 OR S43

75. S7 OR S8 OR S9 OR S10 OR S11 OR S12 OR S13 OR S14 OR S15 OR S16 OR S17 OR S18 OR S19 OR S20 OR S21 OR S22 OR S44 OR S45 OR S46 OR S47 OR S48 OR S49 OR S50 OR S51 OR S52 OR S53 OR S54 OR S55 OR S56 OR S57 OR S58 OR S59 OR S60 OR S61 OR S62 OR S63 OR S64 OR S65 OR S66 OR S67 OR S68 OR S69 OR S70 OR S71

76. S74 AND S75

77. S73 OR S76

78. S72 AND S77

79. S72 AND S77

80. S72 AND S73

81. S72 AND S74 AND S75

82. S80 OR S81 [Limiters - Date Published: 20200101-20221231]

***S.7 - R code to search MedRxiv***

# Loading packages

library(medrxivr)

library(dplyr)

mx_snapshot ()

# Building the search

topic1 <- c("COVID*", "sars-cov-2", "coronavirus") # Combined with Boolean OR

topic2 <- c("vaccin*", "immuni*ation") # Combined with Boolean OR

topic3 <- c("child*", "infant", "newborn", "routine", "schedule*", "BCG", "polio*", "IPV*", "OPV*", "DTP*", "TDAP*", "diphtheria", "pentavalent", "hexavalent", "whooping", "pertussis", "tetanus", "pneumococc*", "PCV*", "meningococc*", "measles*", "MCV*", "mumps", "rubella", "RCV*", "MMR*", "HPV*", "HPV*", "papillomavirus", "rota*", "h*emophilus", "HIB*", "Hep*B", "hepatitis") # Combined with Boolean OR

myquery <- list(topic1, topic2, topic3) # Combined with Boolean AND

# Running the search

results <- mx_search(

data = preprint_data,

query = myquery,

fields = c("title", "abstract", "category"),

from_date = "2020-01-01",

to_date = "2022-02-11",

auto_caps = TRUE,

NOT = "",

deduplicate = TRUE,

report = FALSE )

#viewing results

mx_export(results)
